# Role of breath-hold lung PET in stage IA pulmonary adenocarcinoma

**DOI:** 10.1186/s13244-023-01446-1

**Published:** 2023-05-25

**Authors:** Zhaoping Cheng, Li Chen, Ximing Wang, Ying Wang, Minjie Zhao, Keyu Zan, Wen Liu, Xiao Cui, Leiying Chai, Min Ge, Kun Li, Yanhua Duan

**Affiliations:** 1 Department of Nuclear Medicine, The First Affiliated Hospital of Shandong First Medical University and Shandong Provincial Qianfoshan Hospital, Jinan, 250014 People’s Republic of China; 2 Department of Ultrasound, Shandong Provincial Hospital Affiliated to Shandong First Medical University, Jinan, People’s Republic of China; 3 Department of Radiology, Shandong Provincial Hospital Affiliated to Shandong First Medical University, Jinan, People’s Republic of China; 4 Central Research Institute, United Imaging Healthcare, Shanghai, People’s Republic of China; 5 Department of Radiology, The First Affiliated Hospital of Shandong First Medical University and Shandong Provincial Qianfoshan Hospital, Jinan, People’s Republic of China

**Keywords:** Positron-emission tomography, Breath hold, Lung, Adenocarcinoma

## Abstract

**Background:**

Respiratory motion during PET acquisition may result in image blurring and resolution loss, reduced measurement of radiotracer uptake, and consequently, inaccurate lesion quantification and description. With the introduction of the total-body PET system, short-time PET acquisition is feasible due to its high sensitivity and spatial resolution. The purpose of this study was to evaluate the additional value of 20-s breath-hold (BH) lung PET in patients with stage IA pulmonary adenocarcinoma.

**Methods:**

Forty-seven patients with confirmed stage IA pulmonary adenocarcinoma were enrolled in this retrospective study. All patients underwent a 300-s FB whole-body PET, followed by a BH lung PET. The SUV_max_, TBR of the lesions and the percentage difference in nodule SUV_max_ (%ΔSUV_max_) and TBR (%ΔTBR) between the two acquisitions was also calculated. The lesions were further divided by distance from pleura for subgroup analysis. The lesion detectability on PET images was the percentage of FDG-positive lesions.

**Results:**

Among 47 patients, the BH lung PET images identified all lung nodules, and there was a significant difference in overall nodule SUV_max_ and TBR between BH PET and FB PET (both *p* < 0.01). The %ΔSUV_max_ and %ΔTBR were significantly higher in nodules adjacent to pleura (≤ 10 mm in distance) than those away from pleura (both *p* < 0.05). The lesion detectability of BH lung PET was significantly higher than that of FB PET (*p* < 0.01).

**Conclusion:**

BH PET acquisition is a practical way to minimize motion artifacts in PET which has the potential to improve lesion detection for stage IA pulmonary adenocarcinoma.

**Critical relevance statement:**

BH PET acquisition is a practical way to minimize motion artifacts in PET which has the potential to improve lesion detection for stage IA pulmonary adenocarcinoma.

**Graphical Abstract:**

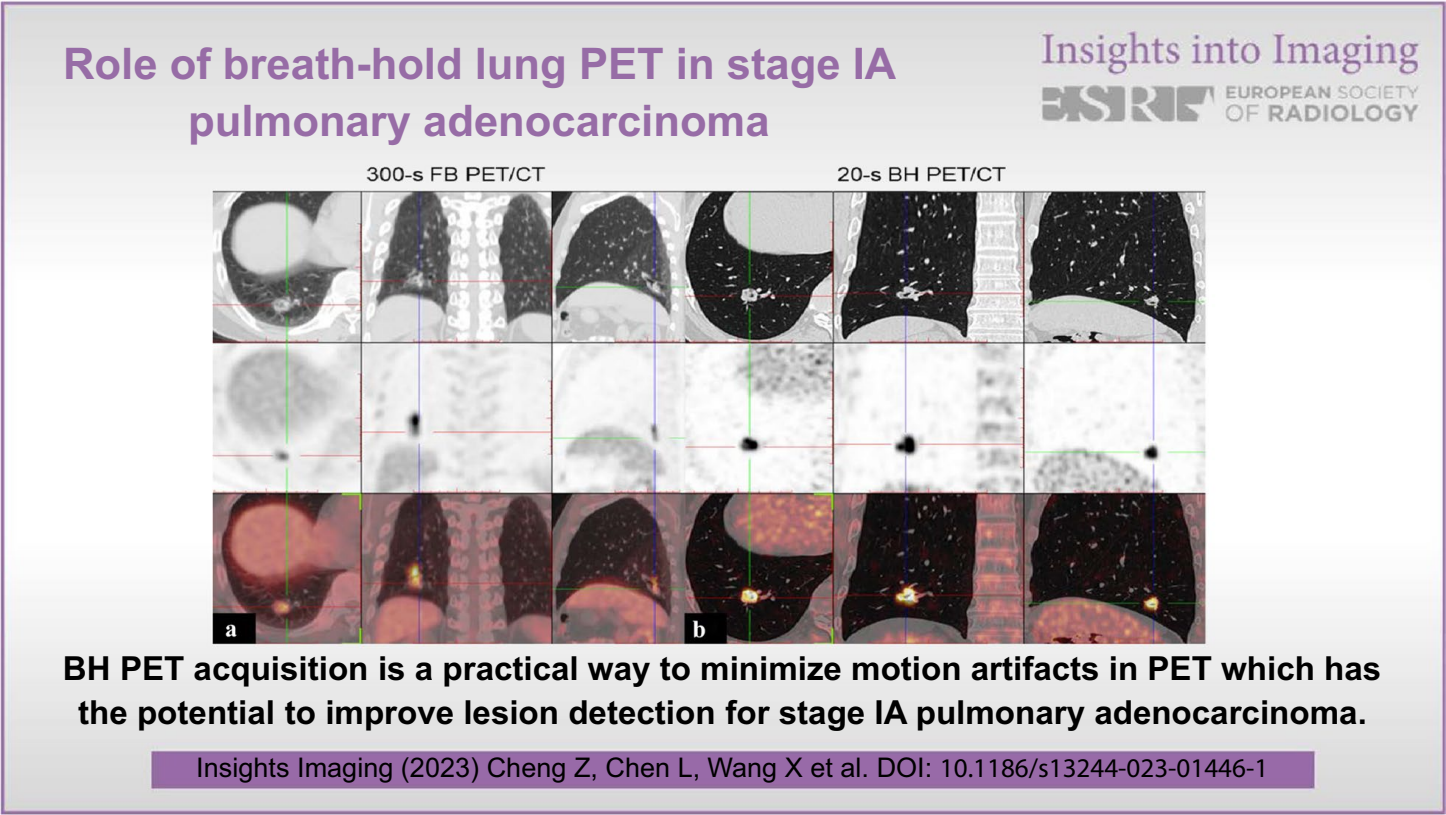

## Key points

- BH lung PET is a complementary approach to minimize motion artifacts.
- BH lung PET can improve the lesion conspicuousness.
- BH lung PET has the potential to improve detectability of pulmonary nodules.

## Background

Mortality of non-small cell lung cancer (NSCLC) can be reduced by identifying lung cancer at an earlier and more treatable stage. Thus, it is important to diagnose the pulmonary nodules at an early stage in clinical routines. The fluorine-18-fluorodeoxyglucose ([^18^F]FDG) PET/CT, combining anatomic data with functional and metabolic information, has been confirmed to provide higher sensitivity, specificity, and accuracy than solely CT for evaluating lung nodules, especially for solid pulmonary nodules (SPNs) and mixed ground-glass nodules (mGGNs) [1, 2]. However, respiratory motion during PET acquisition may result in image blurring and resolution loss, reduced measurement of radiotracer uptake, and consequently, inaccurate lesion quantification and description (in terms of tumor size, location and shape) [3, 4].

In a whole-body PET study, about 15–25 min is required to complete the scan (1–3 min/field of view × 6–8 fields of view). Hence, minimizing respiratory motion artifacts poses a significant challenge in traditional free-breathing (FB) PET [5, 6]. Moreover, the motion problem is more challenging in hybrid PET/CT imaging because of the different respiratory phases between the PET and CT acquisition. Several respiratory gating techniques have been introduced to compensate for respiratory motion artifacts in PET. Hardware-based respiratory gating technique has been used to reduce respiratory motion blurring. However, the installation process is time-consuming and complicated, which involves highly trained personnel. Also, it is based on the external motion information as surrogate parameters of respiratory motion rather than internal organ movements [7]. The data-driven gating (DDG) method has been introduced as an alternative approach that utilizes the motion information extracted from PET raw data. However, the DDG method is also time-consuming because it requires the reconstruction of all gated images and is restricted by the limited spatial resolution of the PET system, particularly in low-dose PET/CT studies [8].

Breath-hold (BH) PET technique has been demonstrated to minimize respiratory motion artifacts and PET/CT image misregistration [9, 10], allowing for better standard uptake value (SUV) measurement for pulmonary nodules and higher diagnostic accuracy for malignant lesions than standard FB PET [9]. Despite previous studies have been performed on PET/CT scanners with a standard axial field of view (AFOV) of ~ 30 cm and limited system sensitivity, the acquired counts in a single breath-hold cycle on such scanners were not adequate, and the image quality and confidence for diagnosis were reduced due to inadequate acquisition [9]. The total-body PET/CT system (uEXPLORER, United Imaging Healthcare), equipped with a 194-cm-long AFOV, can offer significantly improved sensitivity over conventional scanners [11, 12]. The gained sensitivity has been proven and utilized to shorten the acquisition time while maintaining comparable image quality and lesion detectability in oncological studies [13, 14].

We hypothesized that a single 20-s BH PET acquisition with a total-body PET/CT system may provide great potential in early-stage lung cancer assessment. Therefore, this study aimed to (a) evaluate the performance of a single 20-s BH lung PET on stage IA pulmonary adenocarcinoma and (b) explore the contribution of BH lung PET in the characterization of lung nodules.

## Materials and methods

### Patients

**Fig. 1:**
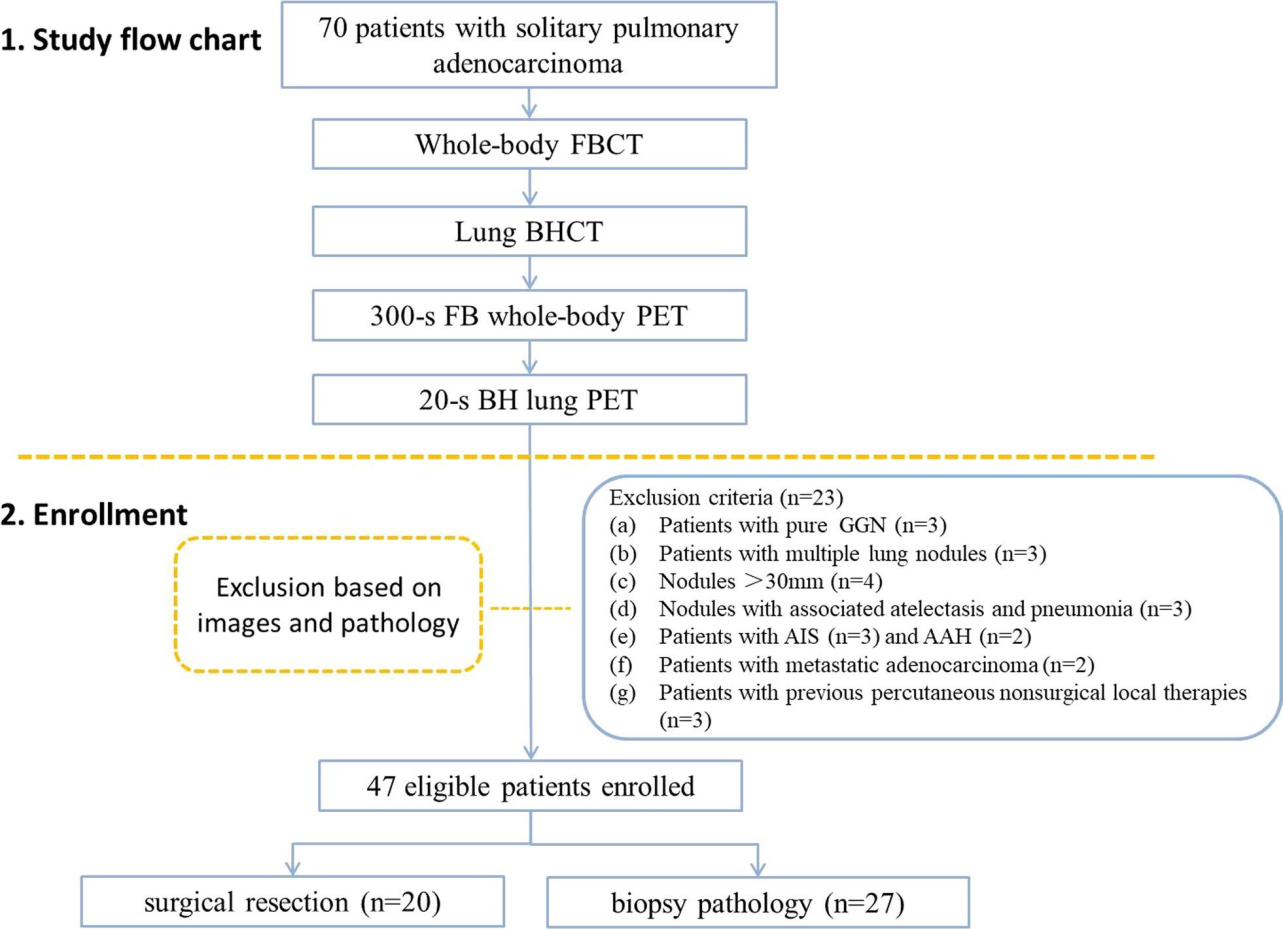
Study flow chart and enrollment

This retrospective study was approved by the institutional review board of our hospital, and written informed consent was obtained from all patients. The study was conducted from May 2020 to December 2020 and recruited a total of 70 patients with solitary pulmonary adenocarcinoma who underwent total-body PET/CT. The inclusion criteria for this study were as follows: (a) solitary solid and part-solid pulmonary nodules with a diameter of less than 30 mm (measured in the CT images using the lung window) without associated atelectasis and pneumonia; (b) pulmonary adenocarcinoma confirmed by surgical or image-guided biopsy pathology; (c) no previous radiotherapy, chemotherapy or other specific oncologic treatments before PET/CT; (d) no history of other malignancies; (e) patients could hold their breath for 20 s during the examination. Twenty-three patients were excluded based on image and pathology (study flow chart and enrollment see Fig. 1), and 47 patients were finally enrolled in this study.

### Total-body PET/CT examination

All patients were asked to fast at least 6 h prior to the PET/CT exam and received a weight-based injection of [^18^F]FDG (2.96 MBq/kg) with blood glucose levels (measured by finger-prick sampling) less than 170 mg/dL. The time interval between FDG administration and the start of PET scanning was approximately 60 min (67.94 ± 6.85 min), and all patients underwent a total-body PET/CT exam.

A low-dose whole-body free-breath CT (referred to as FBCT) and a low-dose deep-inspiration breath-hold thoracic CT (referred to as BHCT) were acquired for attenuation correction and anatomic reference for 300-s free-breath PET (referred to as FB PET) and 20-s BH PET (referred to as BH PET), respectively. The patients were instructed to hold the breath at maximum inspiration for BHCT and BH PET. For the whole-body FBCT and thoracic BHCT, acquisitions parameters were as follows: tube voltage 100 and 120 kV for patients with body mass index ≤ 25 kg/m^2^ and > 25 kg/m^2^, tube current 30 mAs, detector collimation 0.5 mm, gantry rotation time 0.5 s, pitch 0.96, and reconstructed slice thickness 1.5 mm.

FB PET and BH PET imaging were acquired immediately after BHCT scanning. The interval time between the completion of the FB PET scan and the start of the BH PET scan was recorded.

All PET images were reconstructed using the ordered subset expectation maximization (OSEM) algorithm with the following parameters: 3 iterations and 20 subsets, matrix of 192 × 192, slice thickness of 2.89 mm, FOV of 600 mm, Gaussian post-filter with a full width at half maximum of 3 mm, as well as time-of-flight (TOF) and point-spread-function (PSF) modeling. Standard corrections, including attenuation, decay, scatter, random, dead time, and normalization, were performed in PET reconstructions.

### Image analysis

All PET/CT images were anonymous and transferred to a commercial medical image processing workstation (uWS-MI, United Imaging Healthcare) for analysis.

### Quantitative analysis

The quantitative analysis was assessed by a nuclear radiologist with 5-year experience in oncological radiology. For each patient, a 3D spherical volume of interest (VOI) with a diameter of 1 cm was drawn on the homogeneous area of the descending aorta away from the edge and calcification. The SUV_mean_ values of the VOIs were recorded. For the target pulmonary nodules, a VOI was placed with a threshold of 40% of the maximum standard uptake value (SUV_max_) value within the contour margin that can automatically define the boundaries of the nodules. The SUV_max_ and metabolic volume (MTV) of the nodules were obtained. The tumor-to-background ratio (TBR) was calculated by dividing the nodule SUV_max_ by the descending aorta SUV_mean_.

The percentage difference in nodule SUV_max_ and TBR between FB PET and BH PET (referred to as %ΔSUV_max_ and %ΔTBR) were obtained as the following equations [15]:

$$\% \Delta {\text{SUV}}_{\max } = \left( {{\text{SUV}}_{{\max \;{\text{BH}}\;{\text{PET}}}} - {\text{SUV}}_{{\max \;{\text{FB}}\;{\text{PET}}}} } \right)/{\text{SUV}}_{{\max \;{\text{FB}}\;{\text{PET}}}} \times 100$$

$$\% \Delta {\text{TBR}} = \left( {{\text{TBR}}_{{{\text{BH}}\;{\text{PET}}}} - {\text{TBR}}_{{{\text{FB}}\;{\text{PET}}}} } \right)/{\text{TBR}}_{{{\text{FB}}\;{\text{PET}}}} \times 100$$

The diameter (mm), volume (mm^3^), density and distance from pleura (mm) of lesions were measured in BHCT images. The agreement between MTV from PET images and volume from BHCT images was analyzed.

In the subgroups, the correlations between quantitative parameters (SUV_max_, BG-SUV_mean_, TBR and MTV) and lesions’ characteristics (size, density, location and distance to pleura) were analyzed.

Subgroup analyses were performed to investigate the factors of respiratory motion. Lesions were firstly divided into solid nodule (G1) and mGGNs group (G2) by morphological characteristics [3]. Lesions were further divided into D1 (≤ 10 mm), D2 (10–20 mm) and D3 (20–30 mm) groups by longest axis diameter and P1 (≤ 10 mm), P2 (10–20 mm) and P3 (> 20 mm) groups by shortest distance from the center of the lesion to the nearest pleural surface (including diaphragm) [16].

### Lesion detectability

All PET/CT images were visually analyzed in a randomized and unpaired order. Two nuclear radiologists with 10-year experience in interpreting PET images independently identified the nodules by visually checking the suspected lesion uptake (FDG positive findings were defined as lesion uptake above background, and FDG negative findings were defined as lesion uptake at/below background) [15].

### Statistical analysis

Statistical analysis was performed using IBM SPSS software package (version 20.0, IBM Corporation) and MedCalc software (version 13.0.2, MedCalc for Windows).

Patients’ characteristics were compared between subgroups using an independent t-test or Kruskal–Wallis H test. Quantitative parameters were compared among 2 different PET acquisitions using the Wilcoxon matched-pairs signed-ranks test. The correlations between quantitative parameters and lesions’ characteristics were analyzed by Spearman’s rank correlation coefficient. Lesion detectability was compared by the Chi-square test or Fisher exact test. The agreement between MTV and the volume of BHCT images was analyzed using a Bland–Altman plot. A *p* value < 0.05 was considered to indicate a significant difference.

## Results

### Patient characteristics

A total of 47 patients (female/male: 22/25), confirmed with stage IA pulmonary adenocarcinoma by surgical resection (*n* = 20) or image-guided biopsy pathology (*n* = 27), were finally enrolled. All the patients’ characteristics are listed in Table 1.

**Table 1:** Patient characteristics

Parameter	Data
Sex*	
M	25 (53.19)
F	22 (46.81)
Age (years)	62.64 ± 12.66 (36–84)
Weight (kg)	65.06 ± 11.51 (43.0–91.0)
Height (cm)	167.66 ± 7.67 (144–184)
Body mass index (BMI, kg/m^2^)	23.09 ± 3.47 (15.21–29.00)
Blood glucose level (mg/dL)	107.95 ± 19.44 (82.80–144)
Injection dose (MBq)	185.30 ± 35.55 (120.25–291.93)
Injection dose per weight (MBq/kg)	2.85 ± 0.19 (2.34–3.21)
Waiting time (min)	67.94 ± 6.85 (55–79)
Lesion size (mm)	17.79 ± 5.81 (6.32–26.64)
Lesion volume (mm^3^)	2101.73 ± 1531.36
Lesion location*	
Left lung	28 (59.57)
Right lung	19 (40.43)
Upper lobes	29 (61.70)
Middle and lower lobes	18 (38.30)

Unless otherwise indicated, data are means ± standard deviations, with ranges in parentheses

*Data are numbers of patients (percentage)

The average interval time between the completion of the FB PET scan and the start of the BH PET scan was 47.10 ± 3.39 s, ranging from 41 to 56 s.

Nodules were categorized as G1 (*n* = 26) and G2 (*n* = 21) groups by morphological characteristics. Lesions were further divided into D1 (*n* = 9), D2 (*n* = 17), D3 (*n* = 21) groups by lesion size or P1 (*n* = 12), P2 (*n* = 16) and P3 (*n* = 19) groups by distance to pleura for subgroup analysis. There were no significant differences in subgroups regarding the patients’ characteristics (all *p* > 0.05).

### Nodule analysis of FB PET and BH PET

**Fig. 2:**
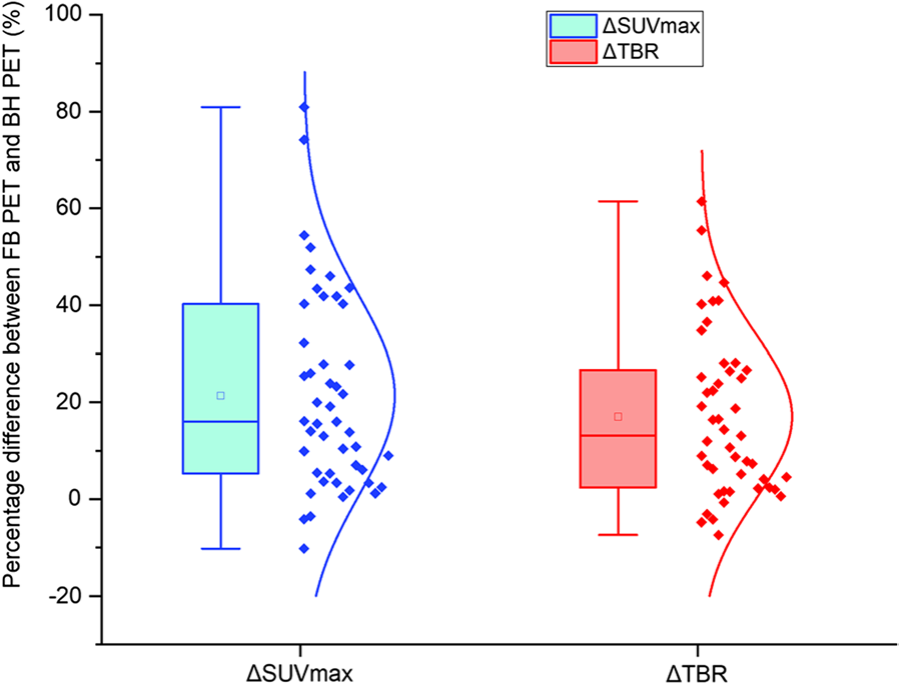
The box plot of the distribution of percent difference between FB PET and BH PET in nodule SUV_max_ and TBR across the entire cohort

**Fig. 3:**
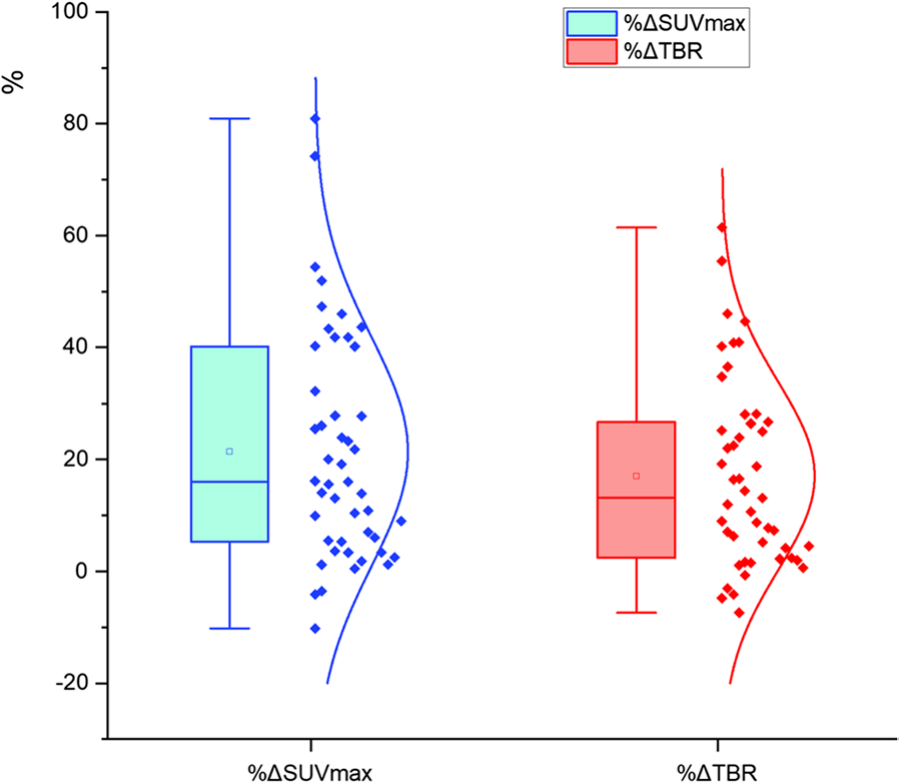
The box plot of the distribution of background (descending aorta) SUV_mean_ between FB PET and BH PET acquisitions across the entire cohort

The BH PET images can improve the lesion conspicuousness compared with the FB PET images. There was a significant difference in overall nodule SUV_max_ and TBR between BH PET and FB PET (both *p* < 0.01), with a mean increase %ΔSUV_max_ of 22.42% (range 80.95 to -10.21%) and %ΔTBR of 21.22% (range 61.47 to -7.37%) (as shown in Fig. 2). The SUV_max_ in 93.62% (44/47) of lesions in BH PET was significantly higher than that in FB PET. Although the descending aorta SUV_mean_ was higher in BH PET than that in FB PET (*p* = 0.016) (as shown in Fig. 3), the TBR in 89.36% (42/47) of lesions in BH PET was yet still significantly higher than that in FB PET. Although the metabolic parameters (SUV_max_) of most lesions were significantly higher in BH PET, the mean MTV of BH PET was significantly reduced (*p* < 0.001).

In the subgroup analysis, the mean and median percentage difference in SUV_max_ (%ΔSUV_max_) and TBR (%ΔTBR) of all subgroups between BH PET and FB PET is summarized in Table 2.

**Table 2:** Results of mean percentage difference in SUV_max_ and TBR in subgroups

	G1	G2	T1a	T1b	T1c	P1	P2	P3
*%ΔSUVmax*								
Mean	21.72%	23.30%	33.64%	18.42%	20.85%	38.75%	20.46%	13.76%
Median	20.02%	18.62%	28.29%	18.42%	18.80%	35.16%	19.05%	10.63%
Range	−3.55 to 54.42%	−10.21 to 80.95%	1.19 to 80.95%	−10.21 to 54.42%	−3.55 to 74.19%	14.04 to 80.95%	0.48 to 54.42%	−10.21 to 40.28%
*%ΔTBR*								
Mean	19.50%	23.36%	37.84%	15.43%	18.80%	36.01%	17.80%	14.77%
Median	30.51%	30.47%	35.92%	28.74%	24.28%	37.50%	19.76%	16.16%
Range	−7.37 to 61.47%	−4.78 to 55.46%	7.05 to 61.47%	−4.78 to 46.05%	−7.37 to 44.69%	1.54 to 61.47%	−4.17 to 46.05%	−7.37 to 36.56%

There were no significant differences between the G1 and G2 groups regarding the mean %ΔSUV_max_ and %ΔTBR in BH PET compared to the FB PET (*p* = 0.848 and *p* = 0.876, respectively). There were no significant differences among groups with different sizes regarding the mean %ΔSUV_max_ and %ΔTBR (all *p* > 0.05). However, the percentage difference of mean SUV_max_ and TBR varied by as much as 15.22–22.41% in the D1 group than D2 and D3 groups.

The %ΔSUV_max_ in the P1 group was significantly higher than those in P2 and P3 groups (*p* = 0.012 and *p* = 0.002, respectively). The %ΔTBR in the P1 group was significantly higher than those in P2 and P3 groups (*p* = 0.034 and *p* = 0.012, respectively), while no significant difference was found between D2 and D3 groups regarding the mean %ΔSUV_max_ (*p* = 0.179) and %ΔTBR (*p* = 0.469). There was a significant inverse correlation between %ΔSUV_max_ and nodule distance to pleura (*p* = 0.004), indicating that the effect of respiratory motion on %ΔSUV_max_ was significantly higher for nodules adjacent to pleura (≤ 10 mm in distance).

### Nodule analysis: volume

**Fig. 4:**
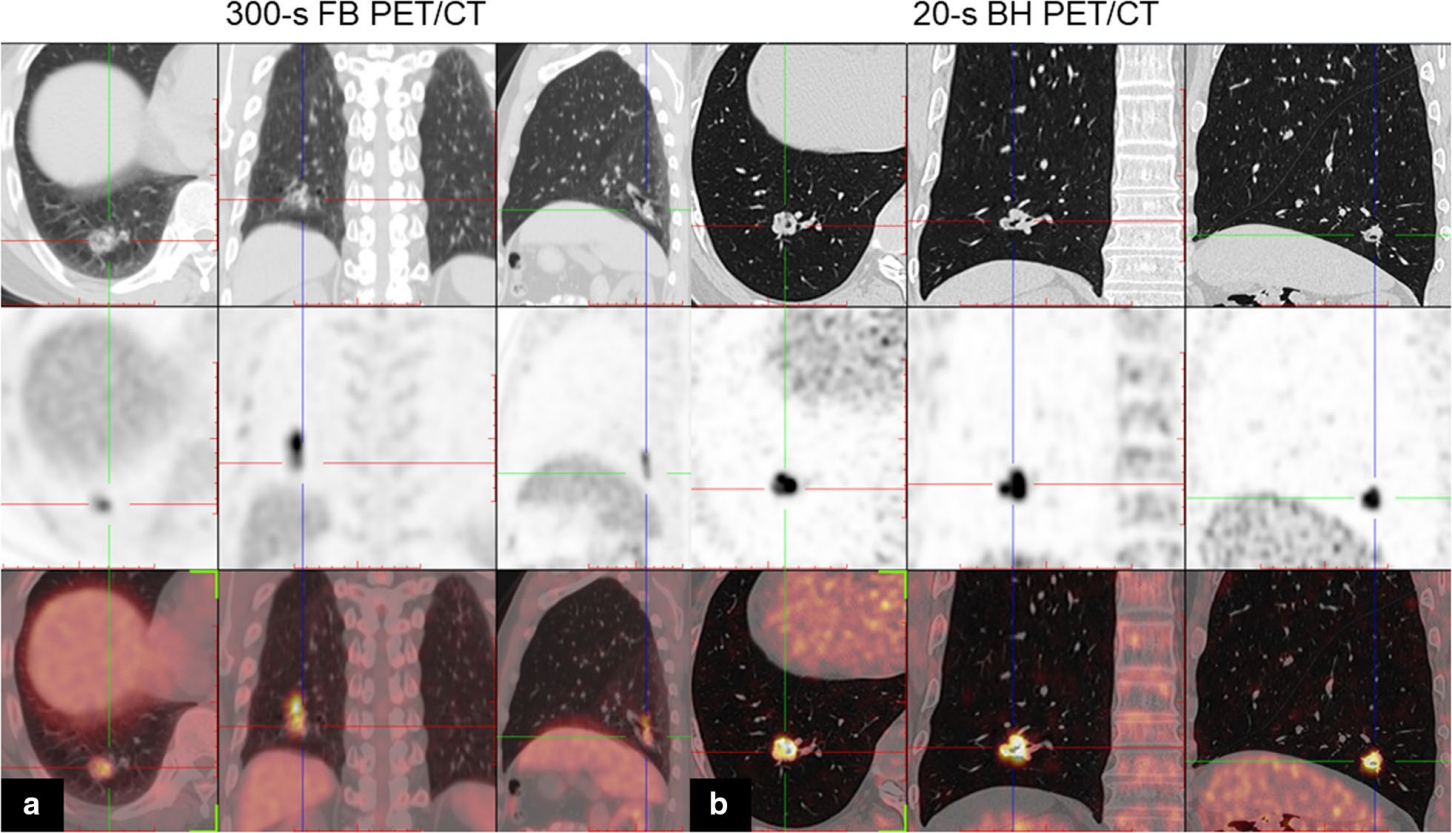
59-year-old man with lung adenocarcinoma. Axial, coronal, and sagittal chest CT (top row), PET (middle row) and PET/CT (bottom row) images of solitary solid lung nodule in the right lower lobe. **a** FB PET/CT; **b** BH PET/CT. Notice how the uptake area appears blurred owing to breathing artifacts in the FB PET image

The mean lesion volume of 47 lesions with BHCT was 2101.73 ± 1531.36 mm^3^. A better agreement was found between the MTV in BH PET and the volume of BHCT, indicating an accurate measurement of BH PET, as shown in Fig. 4.

### Lesion detectability

**Fig. 5:**
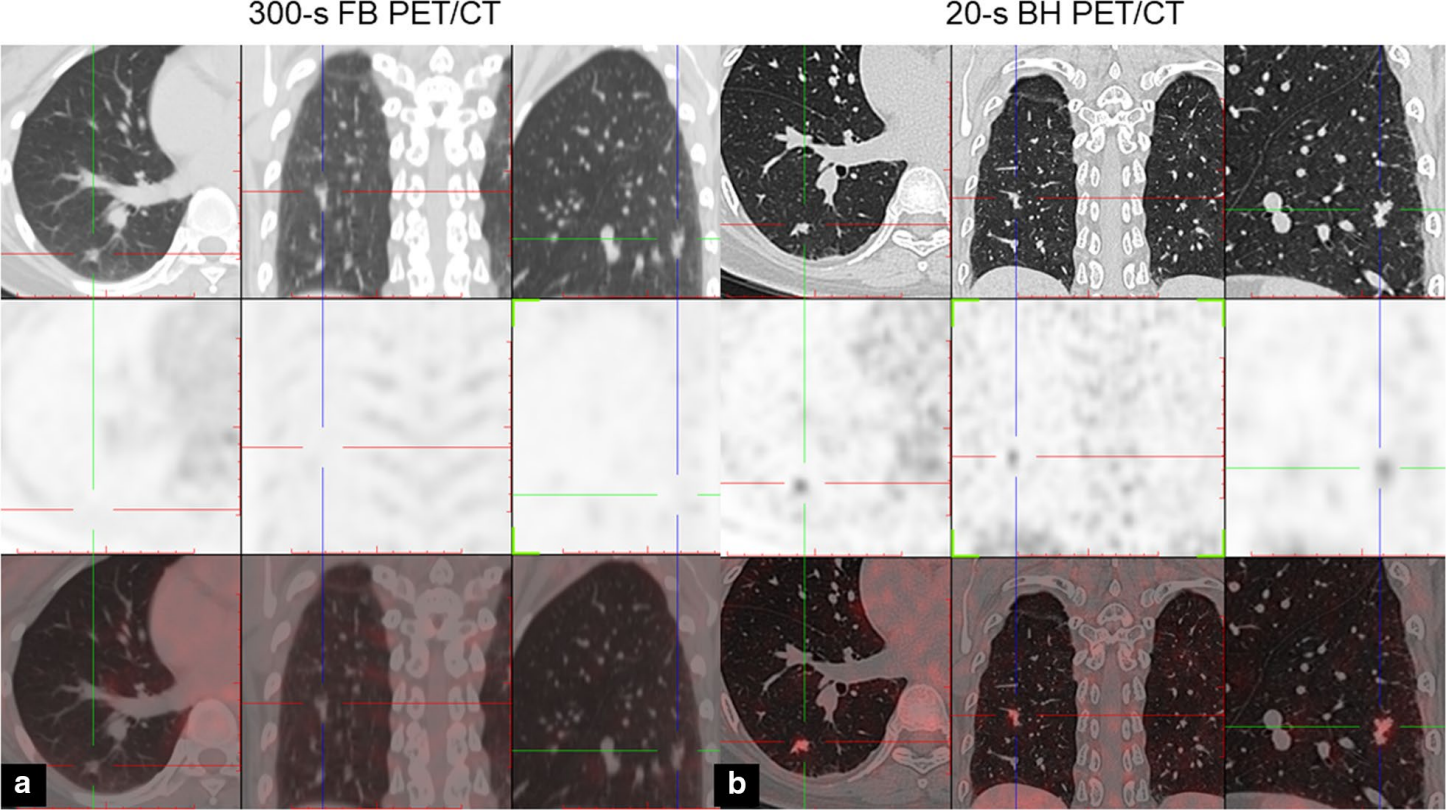
44-year-old woman with lung adenocarcinoma. Axial, coronal, and sagittal chest CT (top row), PET (middle row) and PET/CT (bottom row) images of solitary solid lung nodule in right lower lobe. **a** FB PET/CT; **b** BH PET/CT. Maximum axial diameter of the lesion was 9.2 mm. Lesion was not clearly visualized on FB PET but was conspicuous on BH PET

Of the 47 adenocarcinoma nodules, 45 FDG-positive nodules and 2 FDG-negative nodules were diagnosed by BH lung PET, 42 FDG-positive nodules and 5 FDG-negative nodules were diagnosed by FB PET (Fig. 5).

The lesion detectability of FB PET and BH PET was 89.36% and 95.74%, respectively. The false negative rate in the entire cohort of BH PET was significantly lower than that of FB PET (*p* = 0.009).

## Discussion

This study was designed to assess the added value of a BH PET acquisition compared with a FB PET for stage IA pulmonary adenocarcinoma. The BH PET resulted in reduced breathing-induced artifacts on PET images of the lung. The main findings are as follows: (a) BH lung PET acquisition is a simple and fast method to reduce the blurring of pulmonary nodules caused by respiratory motion; (b) BH lung PET acquisition reveals an increased metabolic activity and the tumor-to-background ratio of nodules, and these increases were significantly higher in nodules adjacent to pleura (≤ 10 mm in distance); (c) BH lung PET acquisition provides a better correlation of the metabolic tumor volume with BHCT volumetric measurements; and (d) BH lung PET acquisition provides a lower false negative rate than conventional FB PET in stage IA pulmonary adenocarcinoma.

[^18^F]FDG PET/CT has become the standard imaging modality for patients with lung cancer. However, its performance in detecting pulmonary nodules is still limited due to the blurring caused by respiratory motion. In routine clinical practice, an additional breath-hold CT is performed to increase diagnostic sensitivity. Efforts have also been made to utilize a high-resolution CT combined with PET to demonstrate superior performance to conventional whole-body PET/CT imaging [17]. However, this method still has to contend with the blurring effect, leading to underestimation of the true concentration of radiotracer uptake, and affecting the activity of lung nodules over a larger area [9]. To avoid motion blurring, amplitude-based and data-driven respiratory gating methods have been introduced [18–20]. However, it has not been widely applied clinically due to the complicated and time-consuming procedure for scanning and data reconstruction. Breath-holding (BH) technique could be considered a fast alternative to respiratory gating. However, BH is challenging in conventional PET as a long acquisition time (1–3 min per bed position in clinical routines) is required. A previous study made an attempt on BH PET using conventional PET scanners to evaluate the feasibility of BH PET acquisition in lung cancer [18], where patients were instructed to hold their breath as long as possible to detect more counts and improve the image quality. Patients whose breath lasted fewer than 29-s were excluded, and the study demonstrated that more than 76.6% (108/141) of the enrolled patients can successfully provide a BH PET acquisition ranging from 30 to 125 s. If the acquisition duration can be further reduced, we believe that the proposed acquisition protocol could increase the probability of completing the BH PET scan. Our data confirmed that a single 20-s BH PET acquisition is feasible for all the enrolled patients with the 194-cm-long AFOV PET scanner while maintaining good image quality. Besides, a shorter breath-holding time could be evaluated for patients with pulmonary dysfunction in further studies. In our study, BH PET offers supplementary advantages over FB PET acquisitions on lung nodules, such as increased SUV_max_ and TBR values, and the degree of this enhancement in our study is consistent with the result from the previous study [18]. Additionally, the injected [^18^F]FDG activity of our study is reduced from 4.64 to 2.85 MBq/kg, hence reducing the radiation dose received by the patients.

In our study, we found that the variability of SUV_max_ of subpleural nodules (≤ 10 mm in distance) is significantly higher than lung nodules further away from the pleural surface (> 10 mm in distance). This result demonstrated that respiratory movement synchronously influences the movement of pulmonary nodules during the breathing cycle, as respiratory movement is one of the main factors that influence the SUV_max_ of FB PET. In our study, there is an interval time between the FB and BH PET acquisitions. Apart from the acquisition mode, the uptake in the latter acquisition should be increased according to the pathological characteristics of the pulmonary adenocarcinoma. We have found the interval time with a limited range (47.1 ± 3.39 s), and thus, we assumed that these factors only cause minimal influence and that the difference in the measurements is mainly from the acquisition mode. Further study should be performed with another enrollment of patients scanned with an alternative order.

The breathing artifact can spread the lung nodules activity over a larger area and generate an overestimation of the MTV of the nodules. In our study, we also found that the lesion volume in the BH PET group was significantly lower than those in the FB PET group and was in good agreement with the standard reference (measured in BHCT). The blur-free images in PET have the potential to define precisely metabolically active tumor volume in tumor radiotherapy planning. Hence, the BH approach, as the easiest way to eliminate motion artifacts during the acquisition of PET/CT images, is highly desirable in clinical routine.

### Limitations

There are several limitations in our study. First, BH PET was only applied to image the lung in this study. The effects of BH PET on the whole body, such as liver, will be discussed in further studies to demonstrate whether BH PET has the potential to improve lesion detection by controlling respiratory motion. Second, only patients with stage IA pulmonary adenocarcinoma were enrolled in this study, which kept the homogeneity of all cases. In future study, different pathologic types of lung nodules will be enrolled to evaluate the differential diagnosis value of BH PET. Third, it was a single-institution respectively study and the sample size was small, and it is necessary to perform a larger, multiple-institution study to improve the reliability and validity of BH PET.

## Conclusions

In conclusion, this study demonstrates BH PET acquisition as a potential way to minimize motion artifacts in PET and to improve small lung nodule detection. BH PET in patients with stage IA pulmonary adenocarcinoma offers supplementary advantages over FB total-body PET acquisitions for assessment of tracer uptake and metabolically active tumor volume in lung nodules, especially for nodules adjacent to pleura (≤ 10 mm in distance). Moreover, BH lung PET acquisition enables a lower false negative rate for stage IA pulmonary adenocarcinoma compared to FB PET.

## Data Availability

The datasets analyzed during the current study are available from the corresponding author upon reasonable request.

## Abbreviations

AFOV: Axial field of view
BH: Breath hold
CT: Computed tomography
DDG: Data-driven gating
FB: Free breathing
FDG: Fluorodeoxyglucose
mGGN: Mixed ground-glass nodule
MTV: Metabolic tumor volume
PET-CT: Positron-emission tomography-computed tomography
SUV: Standardized uptake value
SUV_max_: Maximum SUV
SUV_mean_: Mean SUV
TBR: Tumor-to-background ratio
VOI: Volume-of-interest
